# SUMOylation by Pias1 Regulates the Activity of the Hedgehog Dependent Gli Transcription Factors

**DOI:** 10.1371/journal.pone.0011996

**Published:** 2010-08-11

**Authors:** Barny Cox, James Briscoe, Fausto Ulloa

**Affiliations:** Developmental Neurobiology, Medical Research Council-National Institute for Medical Research, London, United Kingdom; National Cancer Institute, United States of America

## Abstract

**Background:**

Hedgehog (Hh) signaling, a vital signaling pathway for the development and homeostasis of vertebrate tissues, is mediated by members of the Gli family of zinc finger transcription factors. Hh signaling increases the transcriptional activity of Gli proteins, at least in part, by inhibiting their proteolytic processing. Conversely, phosphorylation by cAMP-dependent protein kinase (PKA) inhibits Gli transcriptional activity by promoting their ubiquitination and proteolysis. Whether other post-translational modifications contribute to the regulation of Gli protein activity has been unclear.

**Methodology/Principal Findings:**

Here we provide evidence that all three Gli proteins are targets of small ubiquitin-related modifier (SUMO)-1 conjugation. Expression of SUMO-1 or the SUMO E3 ligase, Pias1, increased Gli transcriptional activity in cultured cells. Moreover, PKA activity reduced Gli protein SUMOylation. Strikingly, in the embryonic neural tube, the forced expression of Pias1 increased Gli activity and induced the ectopic expression of the Gli dependent gene Nkx2.2. Conversely, a point mutant of Pias1, that lacks ligase activity, blocked the endogenous expression of Nkx2.2.

**Conclusions/Significance:**

Together, these findings provide evidence that Pias1-dependent SUMOylation influences Gli protein activity and thereby identifies SUMOylation as a post-translational mechanism that regulates the hedgehog signaling pathway.

## Introduction

The Hedgehog (Hh) family of secreted molecules is crucial during development and adult homeostasis, regulating diverse biological processes comprising cell specification and proliferation (reviewed in [1], [2]). Dysregulation of the pathway is implicated in a range of human diseases that include several congenital syndromes and common cancers proliferation (reviewed in [3], [4]). In vertebrates, Gli proteins, zinc finger transcription factors, which are orthologues of Drosophila Ci, mediate Hh signaling [5]. To date, three Gli proteins, Gli1-3, have been identified. While Gli1 appears to act solely as a transcriptional activator, Gli2 and Gli3 display both transcriptional activator and repressor properties and Gli3 is considered to function mainly as an antagonist of the pathway (reviewed in [6], [7]). Despite the importance of Hh signaling, the mechanisms that regulate Gli activity remain to be fully elucidated. Accumulating evidence suggests that proteolytic processing by the ubiquitin-proteasome system restrains Gli activity and/or promotes transcriptional repressor activity [8]. In the absence of Hh signaling, PKA-dependent phosphorylation of a cluster of serine residues C-terminal to the zinc finger DNA binding domain of Gli2 and Gli3 recruits the βTrCP subunit of the SCF-ubiquitin-ligase complex. Subsequent ubiquitination targets Gli2 and Gli3 to the proteasome [9], [10], [11], [12]. In the case of Gli2, this appears to result in its complete degradation [10], [13]. By contrast, Gli3 is partially processed by the proteasome to generate a C-terminal truncated protein that acts as a transcriptional repressor [12], [14], [15], [16]. Hh signaling inhibits proteolytic processing of both Gli2 and Gli3 and, as a consequence, these proteins accumulate [10], [15], [16]. It is less clear if Hh signaling influences the activity of Gli1, but it is conceivable that regulated ubiquitination also plays a role in the post-translational control of Gli1. Whether additional post-translational mechanisms control the activity of Gli proteins remains an open question.

Small ubiquitin-related modifier (SUMO) are a family of small proteins (∼10 kDa) with a similar structure to ubiquitin [17], [18]. The reversible conjugation of SUMO to lysine residues has been implicated in the regulation of the activity of several proteins (reviewed in [18]). Four SUMO paralogs have been described in mammals (SUMO1-4), of which SUMO1–3 are ubiquitously expressed, while the expression of SUMO4 is more restricted [19] (reviewed in [18], [20]). The process of protein SUMOylation is similar to that of ubiquitination requiring Aos1/Uba2 (an E1 activating enzyme) and Ubc9 (an E2 conjugating enzyme) activity. Additionally, E3 ligases contribute to SUMOylation substrate specificity and efficiency. Three main subtypes of SUMO E3 ligases have been identified: Pias proteins, RanBP2, and Pc2 [18], [20]. However, by contrast to ubiquitination, which tends to promote degradation of target proteins, the impact of SUMOylation on proteins is more diverse and less predictable. SUMO conjugation has been documented to produce changes in cell location, stability or association with other molecules and SUMO conjugation to transcription factors has been reported to both activate and inhibit transcriptional activity (reviewed in [21], [22]).

Here we provide evidence that Gli proteins can be SUMOylated. Moreover, the E3 SUMO ligase, Pias1, and SUMO modification influences the transcriptional activity of Gli proteins. PKA activity appears to negatively regulate Gli protein SUMOylation. Furthermore, experiments in the embryonic neural tube, a tissue in which Hh signaling has a well defined and essential role (reviewed in [23], [24]), suggest that SUMOylation of Gli proteins is required for the activation of a Gli target gene in vivo. Together, these data identify SUMOylation as a previously overlooked post-translational modification of Gli proteins that affects transcriptional activity and raises the possibility that regulated SUMOylation of Gli proteins functions to modulate Gli activity in vivo.

## Results

### Gli Proteins Are SUMOylated on Specific Lysines In Vivo

**Figure 1:**
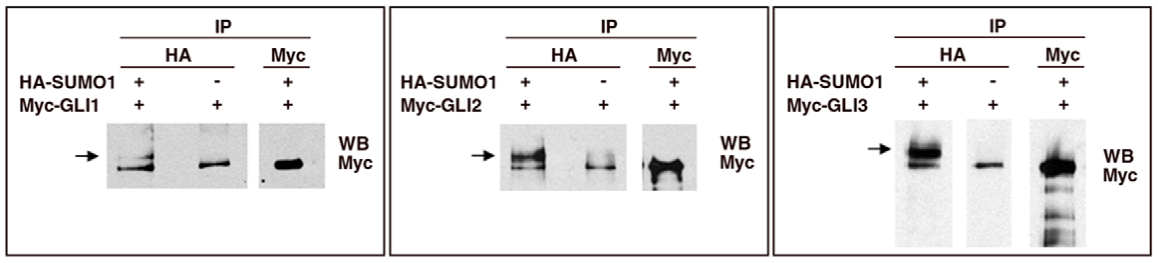
Gli proteins can be SUMOylated. HEK293 cells were co-transfected with constructs encoding HA-SUMO1 and the indicated Myc-tagged Gli proteins. After 36 h, cells protein extracts were obtained and co-immunoprecipitation assays were carried out using antibody recognizing the HA epitope. Then, the co-precipitated material was detected by Western blot using and an anti-Myc antibody (for details see Material and Methods). Bands with slower electrophoretic mobility than native Gli proteins, correspondening to SUMOylated Gli forms, were detected in the co-precipitated material (arrows). In many of our experiments non-modified Gli proteins precipitated with the Protein G beads. This non-specific binding could not be reliably removed in any binding or washing conditions tested. SUMO specific conjugation was therefore analyzed, and confirmed; using specific lysine to arginine Gli mutated forms (see below).

In the course of a two-hybrid screen to identify proteins that interact with Gli3, Pias1- a component of the SUMOylation pathway - was recovered. We could not confirm this interaction by co-immunoprecipitation assays (not shown). However, since Pias1 is reported to act as a SUMO E3 ligase [25], [26] and enzymatic interactions may be hard to demonstrate by co-immunoprecipitation, we sought to test whether Gli proteins were SUMOylated in vivo. Using HEK293 cells, we co-expressed HA-SUMO1 and either Myc-Gli1, Myc-Gli2 or Myc-Gli3. After 36 h, SUMOylated proteins were immunoprecipitated and the presence of Gli proteins in the immunoprecipitated material was assayed by western blot (Fig 1). In extracts of cells co-transfected with HA-SUMO1 and Gli1, Gli2 or Gli3, isoforms of the Gli proteins were detected that migrated slower than predicted by their native molecular weights. These heavier than expected isoforms were only present in samples co-transfected with HA-SUMO1 and were approximately 20–30 kD heavier than the native proteins, consistent with the reported mobility shift resulting from the addition of SUMO [20]. Similar results were obtained using NIH3T3 cells (not shown). We also found that SUMO2 could be conjugated to Gli1 (not shown). Unfortunately, non-specific precipitation of Gli proteins was detected in cell extracts incubated with Protein G beads alone and although various blocking and extensive washing conditions were tested, none was sufficient to reliably eliminate the non-specific precipitation. Nevertheless, the presence of slower migrating bands in each of the extracts from cells co-transfected with HA-SUMO1 and the Gli proteins suggest that SUMO1 can be covalently conjugated to the Gli proteins in vivo.

**Figure 2:**
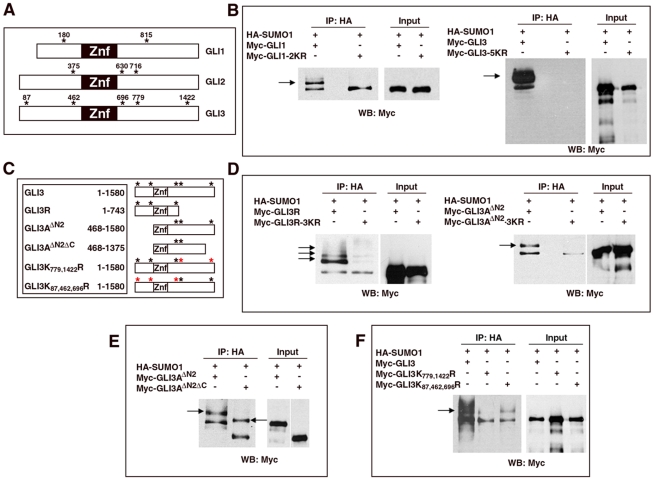
Sites of SUMOylation in Gli proteins. A. SUMOylation consensus sites are present in Gli proteins. The diagram shows the position of the lysine residues where SUMOylation is predicted in human Gli1 (Gli1), mouse Gli2 (Gli2) and human Gli3 (Gli3). B. Co-precipitation assays performed in HEK293 cells co-transfected with HA-SUMO1 and mutated forms of Myc-tagged Gli1 and Gli3 proteins in which all the predicted SUMOylatable lysine residues (see above) have been changed to arginine (Gli1-2KR, Gli3-5KR). SUMOylation of these mutated Gli proteins was markedly diminished or abolished. C. Diagram explaining the structure of the different mutated forms of Gli3 used in Fig. 2D–F. D–F. Co-precipitation assays performed in HEK293 cells co-transfected with HA-SUMO1 and the Myc-tagged mutated forms of Gli3 indicated in C. Arrows indicate sumoylated forms. D. Both Gli3 truncated forms, Gli3R (which lacks most of the carboxy-terminal domain of Gli3), and Gli3A^ΔN2^ (which lacks the N-terminal domain) were SUMOylated in HEK-293 cells. SUMOylation was abolished in Gli3A^ΔN2^-3KR, a form of Gli3A^ΔN2^ where all predicted SUMOylatable lysine residues (see above) were changed to arginine. By contrast, some SUMOylation was still observed in Gli3R-3KR indicating the existence of SUMOylatable sites in this region of Gli3 other than those predicted in A. E. The truncated forms of Gli3, Gli3A^ΔN2^ and Gli3A^ΔN2ΔC^, were SUMOylated in HEK-293 cells. This suggested that the K1422 residue, which is absent in Gli3A^ΔN2ΔC^, is not required for the SUMOylation of Gli3A^ΔN2^. F. SUMOylation of the mutant Gli3K_779,1422_R was markedly reduced in comparison to Gli3K_87,462,696_R and wild type Gli3.

Comparison of the amino acid sequences of the Gli proteins to the SUMOylation consensus motif ΨKXE (where Ψ is an aliphatic branched amino acid, and X any amino acid [27], reviewed in [18]) revealed several potential SUMOylation sites in each protein (Fig 2A). Two consensus sites were present in Gli1 and conserved through the vertebrate lineage. Three conserved consensus sites were present in Gli2. The equivalent sites were also present in Gli3 along with two additional sites (Fig 2A). To corroborate the SUMOylation of Gli proteins, Gli constructs were prepared in which specific lysines were replaced with non-SUMOylatable arginine residues. For these experiments we focused on Gli1 and Gli3. In Myc-Gli1-2KR both lysine 180 and 815 were mutated to arginine and in Myc-Gli3-5KR lysines 87, 462, 696, 779 and 1422 were substituted with arginine. These constructs were then assayed for their ability to conjugate HA-SUMO1 in cells. SUMOylated forms of wild-type Myc-Gli1 and Myc-Gli3 were readily detected in HEK293 cells expressing HA-SUMO1. In contrast neither Myc-Gli1-2KR nor Myc-Gli3-5KR appeared conjugated with SUMO1 (Fig 2B). These data confirm the SUMOylation of Gli1 and Gli3 and suggest that one or more of the mutated lysines represent the major SUMOylation sites.

To establish which of the five mutated lysines in Gli3-5KR are necessary for SUMOylation, we assayed an additional set of Gli3 constructs (Fig 2C). We first examined the SUMOylation of Gli3A^ΔN2^ and Gli3R, these consist of Gli3 lacking the amino-terminal and most of the carboxy-terminal domains, respectively. Both of these constructs were SUMOylated in HEK293 cells suggesting that both the amino and carboxy domains contain SUMOylatable residues (Fig 2D). Moreover, multiple slower migrating bands were apparent upon immunoprecipitation of Gli3R from SUMO1 transfected cells, compatible with the possibility that this region contains more than one SUMOylation site. In contrast to the wild-type versions of Gli3A^ΔN2^ and Gli3R, the equivalent constructs harbouring lysine to arginine substitutes in K696, 779 and 1422 (Gli3A^ΔN2^-3KR) and in K87, 462 and 696 (Gli3R-3KR) showed a marked reduction in SUMOylation (Fig 2D). Immunoprecipitation of Gli3R-3KR revealed a significant reduction but not a complete absence of slower migrating bands. This suggests this region of Gli3 may contain one or more sites in addition K87, K462 and K696 that can be conjugated to SUMO1, albeit inefficiently. Despite the ability of Gli3R to be SUMOylated in these assays, it was notable that when SUMOylation was assayed using full length Gli3 only isoforms that migrated slower than the full length protein were detectable. Thus, even though transfection of full length Gli3 generated significant quantities of processed Gli3, this did not appear to be SUMOylated in vivo (data not shown). These findings suggest that full length but not processed Gli3 is a substrate for SUMOylation, raising the possibility that either the N-terminal of full length Gli3 is not SUMOylated or that SUMOylated Gli3 is not a substrate for proteolytic processing.

No slower migrating bands were detectable in samples of Gli3A^ΔN2^-3KR transfected cells. This indicates that K696, 779 and/or K1422 encompass the major SUMOylation sites in the carboxy-terminal domain of Gli3. We therefore analyzed which residues in Gli3A^ΔN2^ are required for SUMOylation. Gli3A^ΔN2ΔC^, a construct missing the last 205 amino acids of Gli3A^ΔN2^ including K1422, was still SUMOylated suggesting that this lysine is not required for SUMOylation in vivo (Fig 2E). This led us to concentrate on K696 and K779 and analyze their requirement for the SUMOylation of full length Gli3. We generated two constructs Gli3K_87,462,696_R and Gli3K_779,1422_R. In Gli3K_87,462,696_R, K87, K462 and K696 were mutated to arginine while the carboxy lysines K779 and 1422 were left intact. Gli3K_779,1422_R had the reciprocal changes: K779 and K1422 were mutated to arginine and K87, K462 and K696 were left intact. Immunoprecipitation of these constructs from HEK293 cells cotransfected with HA-SUMO1 indicated that Gli3K_87,462,696_R continued to be SUMOylated, however, the amount of SUMOylation was noticeably decreased for Gli3K_779,1422_R (Fig 2F). Together these data suggest that K779 is likely to be the main site of SUMOylation of Gli3. Notably this lysine has also been shown to be ubiquitinated by βTrcp when recruited in response to PKA phosphorylation [12].

### PKA Regulates the SUMOylation of Gli2 and Gli3

**Figure 3:**
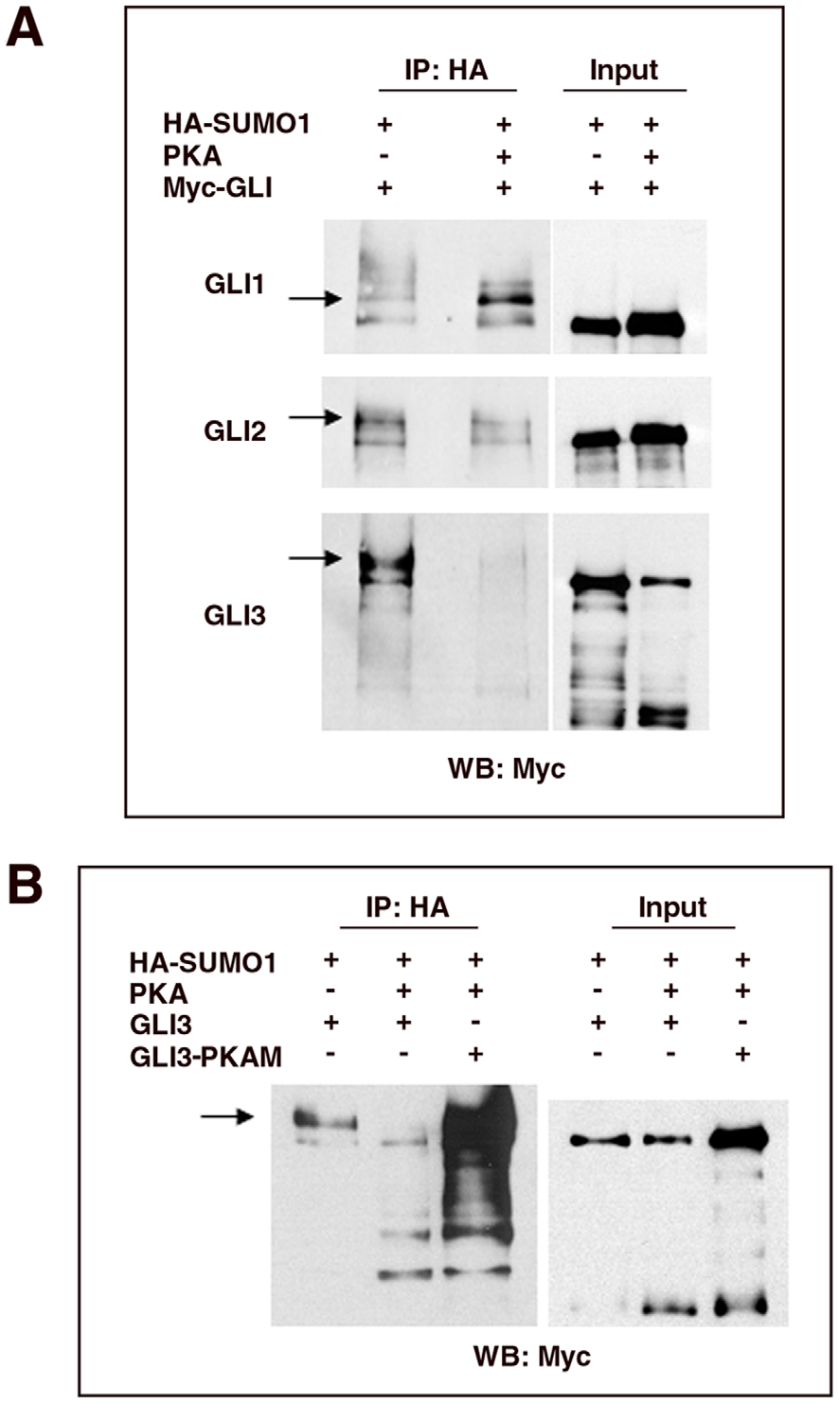
PKA inhibits SUMOylation of Gli2 and Gli3. A. Co-immunoprecipitation assays performed in HEK293 cells co-transfected with HA-SUMO1, the indicated Glis and constitutively active PKA (PKA). Gli2 and Gli3 proteins were less SUMOylated in the presence of constitutively active PKA. B. Co-immunoprecipitation assays performed in HEK293 cells co-transfected with HA-SUMO1, constitutively active PKA, and either a wild type Myc-Gli3 or a mutated form of Myc-Gli3 unable to be phosphorylated by PKA (Gli3-PKAM). Constitutively active PKA did not inhibit the SUMOylation of Gli3-PKAM.

Previously PKA stimulation has been shown to regulate the transcriptional activity of Gli2 and Gli3 by inducing the hyperphosphorylation of Gli2 and Gli3 [10], [16]. To determine whether PKA could also influence the SUMOylation status of Gli1, Gli2 and Gli3 we performed immunoprecipitations from cells co-transfected with HA-SUMO1 and an expression vector encoding a constitutive active PKA. The expression of PKA appeared to have a relatively minor effect on the SUMOylation status of Gli1, a change in the proportions of the slower migrating, SUMO conjugated isoforms, was observed in some experiments, but SUMOylated forms of Gli1 remained readily detectable (Fig 3A). Strikingly, however, compared to cells that had not received the PKA vector, the level of Gli2 and Gli3 SUMOylation was markedly reduced in the presence of activated PKA (Fig 3A). The reduction in SUMO1 conjugated forms of Gli2 could not be accounted for by a decrease in the level of Gli2 protein because the reduction in SUMOylated form of Gli2 was much greater than any reduction in the native protein level. Similarly, PKA stimulation increased the ratio of repressor to full length isoforms of Gli3 as expected, but the reduction in the level of SUMOylated Gli3 was much greater than any reduction in Gli3 protein levels. As noted above there was no evidence that the processed form of Gli3 was SUMOylated. Moreover, the lack of inhibition of Gli1 SUMOylation suggested that the effect of PKA on the SUMOylation of Gli2 and Gli3 was specific.

To test whether the regulation of Gli3 SUMOylation by PKA depends on the direct phosphorylation of Gli3, we took advantage of a construct, Gli3-PKAM, in which the 6 serines phosphorylated by PKA are mutated to alanine [16]. Mutation of these sites completely abrogates the ability of PKA to phosphorylate and regulate activity of Gli3 [16]. In marked contrast to wild-type Gli3, PKA did not inhibit the SUMOylation of Gli3-PKAM (Fig 3B). Indeed, in cells expressing Gli3-PKAM and constitutive active PKA, larger than expected quantities of SUMOylated Gli3 were recovered, suggesting that mutation of these serines in Gli3 may enhance SUMOylation. Together these data provide evidence that PKA regulates SUMOylation of Gli2 and Gli3. Moreover, the data argue against an indirect effect of PKA on SUMOylation of Gli3 and suggest that the amount of SUMO1 conjugated to Gli3 is inversely related to the phosphorylation of the identified PKA sites in Gli3.

### Pias1 enhances the transcriptional activity of Gli proteins and is required for the regulation of a Gli target gene

**Figure 4:**
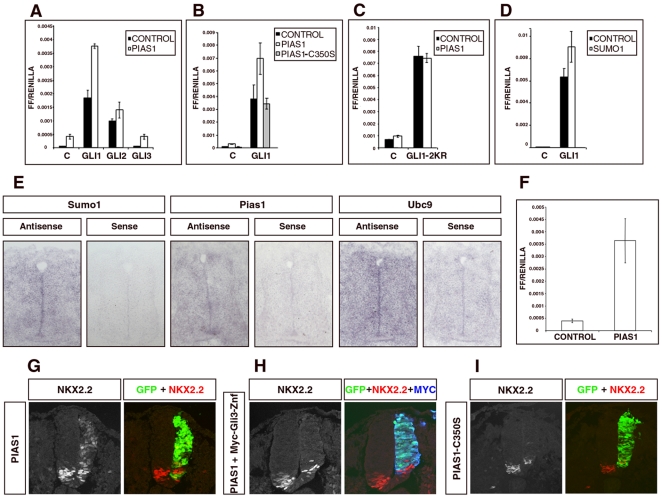
Pias1/SUMO enhances Gli activity. A–D. Pias1/SUMO enhances Gli activity in cultured cells. Luciferase assays in NIH3T3 cells using a GBS-firefly luciferase reporter and CMV-renilla luciferase for normalization. cDNAs encoding the indicated proteins and the appropriate empty vectors – to ensure equal amounts of DNA in each condition – were cotransfected with reporter and normalization plasmids. Firefly and renilla luciferase activities were assayed after 24 h. The forced expression of Pias1 increased GBS reporter activity (A), particularly in cells co-transfected with Gli1 or Gli2. By contrast, the forced expression of a mutated form of Pias1 that lacks E3-ligase activity (Pias1-C350S) did not affect Gli1 activity (B). Consistent with this, the activity of a non-SUMOylatable form of Gli1 (Gli1-2KR) was not affected by the forced expression of Pias1 (C), and the over-expression of SUMO1 enhanced Gli1 activity although at a lesser extent that Pias1 (D). E. SUMO pathway components are expressed in the developing chick neural tube. Transverse sections at the thoracic level of chick embryos. The expression of the indicate genes was detected by in situ hybridization. Sense controls for each probe were used to test specificity. F. Pias1 enhances Gli activity in embryos. In ovo luciferase assay. HH st 11–12 chick embryos were co-electroporated with the GBS-firefly luciferase reporter and CMV-renilla luciferase normalization plasmid together with Pias1 in pCAGGS vector or an empty pCAGGS vector. The levels of firefly and renilla luciferase were measured 24 h after the electroporation. G–I. Forced expression of Pias1 induces the ectopic expression of Nkx2.2 in a Gli activity dependent manner. G. HH st chick embryos were electroporated with pCAGGS-Pias1 and assayed 24 h later by fluorescence confocal microscopy for the expression of the Gli responsive gene Nkx2.2 (red). Electroporated side is on the right and electroporated cells are indicated by GFP immunofluorescence (green). Ectopic induction of Nkx2.2 was observed in electroporated cells. H. Co-electroporation of Pias1 with a construct encoding for the zinc finger domain of Gli3 (Myc-Gli3-Znf) (blue), which inhibits endogenous Gli activity, blocked the ectopic expression of Nkx2.2 (red). I. Electroporation of the mutant Pias1-C350S, which lacks E3-ligase activity, was unable to induce an ectopic expression of Nk2.2 (red). Moreover, an inhibition of the expression of Nkx2.2 in its endogenous domain was observed.

The evidence that Gli proteins can be conjugated by SUMO prompted us to examine whether the SUMOylation pathway, and in particular Pias1, affected the transcriptional activity of the Gli proteins. A consensus binding site has been defined for the Gli proteins and a reporter (GBS-Luc) consisting of 8 concatemerized sites provides a reliable assay for Gli transcriptional activity [28]. We first tested the effect of Pias1 on Gli activity in NIH3T3 cells. Transfection of a Pias1 expression construct resulted in a significant increase in GBS-Luc activity, suggesting that promoting SUMOylation is sufficient to augment Gli activity in cells (Fig 4A). Moreover, Pias1 expression also increased the activity of transfected Gli1, and Gli2 in NIH3T3 cells. Since Pias1 strongly affected the transcriptional activity of Gli1, we focused on Gli1 for the next assays. To determine if the Pias1 effect on Gli proteins was related to their SUMOylation, we analyzed the effect of a mutant form of Pias1 lacking its E3 ligase activity, Pias1-C350S [29], [30], on Gli1 transcriptional activity. By contrast with the wild type form, Pias1-C350S mutant did not affect Gli1 activity (Fig 4B), indicating that the effect of Pias1 on Gli proteins activity was dependent on its ability to conjugate SUMO. Furthermore, Pias1 did not modify the activity Gli1-2KR, the version of Gli1 lacking SUMOylation sites (Fig 4C). Consistent with these data, transfection of SUMO1 into NIH3T3 increased the activity, albeit to a lesser extent that Pias1, of Gli1 in NIH3T3 cells (Fig 4D). Together these data indicate that Pias1/SUMO conjugation enhance the transcriptional activity of Gli proteins.

To investigate whether Pias1/SUMOylation also influences Gli activity in tissue that responds to Hh signaling, we turned to the chick neural tube. We confirmed that SUMO1, PIAS1 and Ubc9 are ubiquitously expressed in the neural tube of chick embryos (Fig 4E). We first tested whether Pias1 increased Gli activity in neural cells in vivo. We introduced, by in ovo electroporation, Pias1, GBS-Luc and normalization plasmids into HH st. 11 neural tubes and assayed luciferase activity 24 h later. Consistent with the data from NIH3T3 cells, expression of Pias1 in the neural tube induced a marked increase in the levels of Gli activity, compared to controls (Fig 4F). We next assayed the effect of Pias1 on the expression of the Nkx2.2 homeodomain transcription factor. This gene is expressed in the ventral neural tube in response to Shh signaling and its induction depends on a regulatory element that directly binds Gli proteins [31], [32], [33]. Consistent with a positive role for SUMOylation on Gli activity, in ovo electroporation of Pias1 induced the ectopic expression of the Nkx2.2 throughout the dorsal ventral axis of the neural tube (Fig 4G). To verify that the ectopic induction of Nkx2.2 expression by Pias1 was dependent on endogenous Gli transcriptional activity we took advantage of Gli3-Znf. This construct consists of only the DNA-binding zinc finger domain of Gli3, which dominantly blocks endogenous Gli activity, and consequently inhibits the Shh dependent induction of Nkx2.2 [34]. In ovo expression of Pias1 together with Gli3-Znf, blocked the ectopic induction of Nkx2.2 expression indicating that the Pias1 induction of Nkx2.2 is dependent on Gli activity (Fig 4H). Finally, to verify that the effect of Pias1 on Nkx2.2 expression was dependent on its E3 SUMO ligase activity, we used Pias1-C350S, which lacks ligase activity. In ovo electroporation of Pias1-C350S was not sufficient to induce ectopic Nkx2.2 expression confirming the Pias1 induction of Nkx2.2 expression depends on SUMO conjugating activity. Furthermore, expression of Pias1-C350S in ventral regions of the neural tube, which would normally express Nkx2.2 in response to Shh signaling, blocked the induction of endogenous Nkx2.2. This suggests that Pias1-C350S can act as a dominant negative [30] to inhibit endogenous Pias1 E3 ligase activity and thereby inhibit Nkx2.2 induction. Together these data indicate that Pias1, through its SUMO ligase activity enhances Gli activity in the neural tube and is required for the induction of a Shh responsive gene.

## Discussion

In this report we provide evidence that Gli proteins are targets of SUMOylation and that Pias1, in a manner dependant on its ability to conjugate SUMO, affects the transcriptional activity of Gli proteins in both cultured cells and in embryos. Each of the Gli proteins has several phylogenetically conserved consensus motifs for SUMOylation and we provide evidence that SUMO1 can be conjugated to the lysines in several of these motifs. The addition of SUMO to Gli proteins appears to boost their transcriptional activating function and this enhancement appears to be necessary for the induction of at least one known target of Gli proteins, Nkx2.2, in the developing neural tube [33], [35], [36], [37]. Consistent with these data, PKA activity, which negatively regulates Gli2 and Gli3, appears to block the SUMOylation of Gli2 and Gli3. Together these data identify SUMOylation as a post-translational modification of Gli proteins that influences the activity of this important family of transcription factors.

SUMOylation appears to correlate with, and to increase, the transcriptional activating function of Gli proteins. SUMOylation of transcription factors is most frequently associated with transcriptional repression [38], however SUMOylation has previously been shown to increase the activity of several transcription factors including p53 and Tcf4 (reviewed in [22]). Our data therefore add to the evidence that SUMOylation can have positive as well as inhibitory effects of the activity of transcription factors. Although we have been unable to detect SUMOylation of endogenous Gli proteins, due to the lack of appropriate reagents, the evidence supports a model in which SUMOylation can regulate endogenous Gli activity in a physiological context. Importantly, SUMO E3 ligase, Pias1, enhances Gli activity in cultured cells and in embryos and this appears to be a consequence of its ability to catalyze the covalent addition of SUMO to Gli proteins (Fig 4). First, a point mutant of Pias1 that lacks ligase activity does not affect Gli1 activity. Second, Pias1 does not affect the activity of a form of Gli1 in which the SUMOylation sites have been mutated to arginine, a residue incapable of SUMO conjugation. Third, over-expression of SUMO1 in cells also increases the activity of Gli1. Together these data favour the idea that direct SUMOylation of a Gli protein alters its transcriptional activity and argue against models in which the effect of SUMOylation is indirect, mediated through the regulated SUMOylation of other proteins. These data also rule out the possibility that the effects of Pias1 on Gli proteins are due to an E3-ligase independent activity of Pias1. Nevertheless, we have not consistently found an increase in SUMOylation levels of Gli proteins in cells transfected with Pias1. However, our experiments relied on the overexpression of tagged SUMO1 and this might make it difficult to detect any effect of increased Pias1 expression on the SUMOylation. How SUMO conjugation contributes to an increase of Gli activity remains unresolved. Although we have not obtained consistent evidence of an increase in the nuclear localization or stability of Gli proteins in the presence of Pias1 (data not shown), we cannot rule out the possibility that changes in the location or stability of a small yet crucial pool of Gli proteins is responsible for the observed effects. Moreover, we do not exclude the possibility that other components of the Hh signaling pathway might also be susceptible to SUMO regulation. In this regard it is notable that Pias1 has been shown to interact with Supressor of fused (Sufu) [39], a negative regulator of the Hh pathway that interacts with Gli proteins [40], [41], [42], [43], [44]. It is possible therefore that sumoylation regulates the Hh pathway at multiple levels.

The mutational analysis of Gli proteins suggest suggests several lysines are potentially conjugated to SUMO. Whether there is a hierarchical relationship between these sites and whether an individual Gli protein harbours multiple SUMOylations is not known. Moreover, the presence of multiple distinct SUMO proteins within cells and the potential for some of these forms to generate polySUMO conjugates [18] raises the possibility that a “SUMO” code exists. This code might result in distinct combinations SUMO modifications at different sets of lysine residues of Gli proteins with each combination resulting in a different effect on the activity of the Gli protein. Further analysis will be required to determine if different patterns of SUMOylation are found on different Gli proteins and to resolve whether these elicit different effects on Gli activity.

PKA is a well established negative regulator of Hh signaling that directly phosphorylates Gli2 and Gli3 to promote their processing by the proteasome [10], [16]. Our data indicate that PKA activity also antagonizes SUMOylation of Gli2 and Gli3. We provide evidence that this antagonism is a consequence of PKA phosphorylation of Gli proteins (Fig 3A, 3B) and not an indirect effect of PKA on the SUMOylation or proteasome machinery. It was also notable that little, if any, SUMO conjugated partially processed Gli3 protein was observed. Together these data suggest that PKA dependent processing of Gli proteins and SUMOylation of Gli proteins are mutually exclusive. In contrast to Gli2 and Gli3, the SUMOylation of Gli1 was less affected by PKA activity. Differences in the distance between the PKA phosphorylation sites and the SUMOylatable lysines in Gli1 and Gli2/3, might account for this observation. Irrespective of the explanation, the data further support the contention that the reduced SUMOylation of Gli2 and Gli3 in the presence of activated PKA is a specific and direct effect.

As Pias1, like other SUMO pathway components, is expressed in the developing neural tube (Fig 4E), it is conceivable that it is capable of regulating the activity of Gli proteins within the neural tube. Here we provide evidence that Pias1 can increase Gli transcriptional activity within the neural tube of chick embryos (Fig 4F). Strikingly, Pias1 can induce ectopic expression of Nkx2.2, a homeodomain transcription factor (Fig 4G), the activation of which has been shown to require high levels of Gli activity [35]. Conversely, inhibition of Pias1 activity blocks Nkx2.2 expression. Taken together, our results suggest a scenario in which Gli proteins require SUMOylation in order to achieve the highest levels of activity necessary to induce the most ventral cell identities within the neural tube.

The finding that Pias1, through SUMOylation, regulates Gli activity within the neural tube raises the possibility that patterning throughout the neural tube may be affected by the SUMOylation machinery. As Pias1 and SUMOylation have been shown to be involved in the regulation of members of Wnt and BMP pathways in other systems [45], [46] and Wnt and BMP signaling, together with Shh, play fundamental roles in the control of cell fate specification and proliferation within the neural tube [47], [48], [49], it is possible that Pias/SUMO regulates the intrinsic activity of proteins that define cell identity, namely members of the homeodomain and basic helix-loop-helix protein families. The extent of the role of SUMO conjugation within the neural tube system remains unknown and will require further investigation.

## Materials and Methods

### Constructs

Gli constructs were designed using human Gli1 and Gli3 and mouse Gli2. All Glis were fused to 6X Myc tag at the N terminus and expressed from the bicistronic expression vector pCAGGS-IRES-nlsGFP [50]. Gli3-Znf consists of aa 477–645 of hGli3, Gli3R consists of aa 1–743 of hGli3, Gli3A^ΔN2^ aa 468–1595 of hGli3 and Gli3A^ΔN2ΔC^ consists of aa 468–1375 of hGli3. All lysine to arginine mutants were generated using a Stratagene Mutagenesis kit according to manufacturer's instructions. Human Gli3 PKA mutant [16] was provided by from P. Beachy (Howard Hughes Medical Institute, Maryland, USA). Human Sumo1 (kindly provided by R. Hay) was fused to an N terminal HA tag, Pias1 and Pias1 C350S mutant have been described [29]. For electroporation experiments Pias1 and Pias1 C350S were subcloned in pCAGGS-IRES-nlsGFP vector. Constitutively active PKA subunit was as described [51].

### Cell Culture and Transfection

HEK293 and NIH 3T3 cells were grown in Dulbecco's modified Eagle's medium (GibcoBRL) supplemented with 10% (vol/vol) fetal calf serum or newborn calf serum, respectively, and cultured at 37°C in 5% (vol/vol) CO_2_ in air.

All cells were transiently transfected using Lipofectamine Plus reagent (Invitrogen) following the manufacturer's instructions.

### Luciferase assay

Luciferase measurements were performed with the Dual-Luciferase Reporter Assay System (Promega) according to the manufacturer's instructions.

### Chick in ovo electroporation

All constructs were electroporated into the neural tube of HH st 10–12 [52] chick embryos [53]. After 24 h, embryos were fixed and processed for immunodetection as described previously [53]. In ovo luciferase assay was performed as previously described [35].

### Coimmunoprecipitation

Transfected HEK293 and NIH 3T3 cells were lysed with lysis buffer (0.15M Tris-HCL, pH6.7, 5% sodium dodecyl sulfate (SDS), and 30% glycerol) and diluted 1∶10 in PBS-0.5% Nonident P-40 (NP-40) plus complete protease inhibitor (Roche). Cell lysates were precleared rotating at 4°C for 1 h with prepared Protein G-Sepharose (Roche). Following removal of preclearing sepharose, protein extracts were then incubated rotating 4°C for one hour with 5 µl of mAb anti-Myc antibody 9B11 (Cell Signaling) or 10 µl of rat Ab anti-HA antibody 3F10 (Roche). Protein G-Sepharose was then added to each sample followed by continued rotation at 4°C for a further 3 h. Beads were then washed four times with PBS-0.5% NP-40 plus complete protease inhibitor and once with 10 mM Hepes/NaOH pH7.0 followed by addition of 30 µl of 2x SDS sample buffer. The immunoprecipitated material was then fractioned by SDS-PAGE, transferred to a nitrocellulose membrane, and detected by immunoblot with the mAb 9E10 (Santa Cruz) to detect the Gli myc epitope or the Ab 3F10 to detect the Sumo HA epitope.

### Immunohistochemistry and in situ hybridization histochemistry

Immunohistochemical localization of proteins was performed as described [53], [54]. mAb mouse Nkx2.2 (74.5A5) antibody came from Developmental Studies Hybridoma Bank (University of Iowa). mAb anti-Myc 9B11 was purchased from Cell Signaling. Sheep GFP antibody (4745–1051) was purchased from Biogenesis. Images were collected on a Leica TCS SP2 confocal microscope. In situ hybridization was performed as described [55] using probes from chick EST clones of Sumo1, Pias1 and Ubc9.
